# Effects of long-term salicylate administration on synaptic ultrastructure and metabolic activity in the rat CNS

**DOI:** 10.1038/srep24428

**Published:** 2016-04-12

**Authors:** Bin Yi, Shousen Hu, Chuantao Zuo, Fangyang Jiao, Jingrong Lv, Dongye Chen, Yufei Ma, Jianyong Chen, Ling Mei, Xueling Wang, Zhiwu Huang, Hao Wu

**Affiliations:** 1 Department of Otolaryngology Head and Neck Surgery, Xinhua Hospital, Shanghai Jiaotong University School of Medicine, Shanghai China; 2 Laboratory of Auditory Neuroscience, Ear Institute, Shanghai Jiao Tong University School of Medicine, Shanghai China; 3 Shanghai Key Laboratory of Translational Medicine on Ear and Nose Diseases, Shanghai China; 4 Department of Otolaryngology Head and Neck Surgery, First Affiliated Hospital of Zhengzhou University, Zhengzhou, Henan Province China; 5 PET Center, Department of Nuclear Medicine, Huashan Hospital, Fudan University, Shanghai China; 6 Department of Nuclear Medicine, Xinhua Hospital, Shanghai Jiaotong University School of Medicine, Shanghai China

## Abstract

Tinnitus is associated with neural hyperactivity in the central nervous system (CNS). Salicylate is a well-known ototoxic drug, and we induced tinnitus in rats using a model of long-term salicylate administration. The gap pre-pulse inhibition of acoustic startle test was used to infer tinnitus perception, and only rats in the chronic salicylate-treatment (14 days) group showed evidence of experiencing tinnitus. After small animal positron emission tomography scans were performed, we found that the metabolic activity of the inferior colliculus (IC), the auditory cortex (AC), and the hippocampus (HP) were significantly higher in the chronic treatment group compared with saline group (treated for 14 days), which was further supported by ultrastructural changes at the synapses. The alterations all returned to baseline 14 days after the cessation of salicylate-treatment (wash-out group), indicating that these changes were reversible. These findings indicate that long-term salicylate administration induces tinnitus, enhanced neural activity and synaptic ultrastructural changes in the IC, AC, and HP of rats due to neuroplasticity. Thus, an increased metabolic rate and synaptic transmission in specific areas of the CNS may contribute to the development of tinnitus.

Tinnitus is the perception of sound in the absence of external auditory stimuli and affects approximately 10% to 15% of the population^1^,^2^,^3^. Tinnitus can affect every aspect of daily life and has a great negative effect on the quality of life^4^,^5^,^6^. Tinnitus can arise from damage at any level of the auditory pathway (cochlear or acoustic nerve or central auditory pathways), but most cases are caused by cochlear damage. These lesions can result in abnormal neuronal activity in the central auditory pathways^1^,^4^,^7^. Increased spontaneous firing rate and/or neural synchrony of neurons in the central auditory system are two of the possible mechanisms for the neural substrate of tinnitus^2^. However, there are many questions unanswered and none of the mechanisms has yet been confirmed. To date, an increasing number of tinnitus studies have focused on changes related to central auditory neuroplasticity^7^,^8^,^9^.

Aspirin, a common salicylate drug that is frequently used in clinical settings, has the potential to cause reversible tinnitus and sensorineural hearing loss. Salicylate-induced tinnitus in rats is a popular animal model for the study of tinnitus^8^,^9^,^10^,^11^,^12^. Many previous studies have indicated that the most substantial pharmacological and pathological effects of salicylate that impact the auditory pathway might be generated at the cochlear level and may develop in the central nervous system (CNS)^8^,^13^,^14^,^15^,^16^. Cochlear abnormalities have been suggested as the trigger of tinnitus, in which altered sensory input is thought to be continuously transmitted from the cochlea to the central auditory pathway. The subsequent cascade of neural alterations in the central auditory system is more likely to maintain the condition^2^. In our previous studies, we developed an animal model of tinnitus by administering salicylate long-term; such long-term treatment progressively raised the amplitude of distortion products of otoacoustic emissions (DPOAEs)^14^, which are mainly caused by increased outer hair cell (OHC) electromotility^17^, enhanced the average spectrum of electrophysiological cochleoneural activity (ASECA)^11^,^18^, and also induced changes to the synaptic ultrastructure of the dorsal cochlear nucleus (DCN)^13^,^19^. Functional reorganization of the central auditory system following peripheral auditory system deafferentation has also been previously reported^15^. We believe that in the tinnitus-centralization mechanism^7^, tinnitus may originate at the cochlear level and develop in the CNS, resulting in neuroplasticity-mediated changes in the synaptic structure and increased metabolic rate of the cerebral auditory areas, such as the inferior colliculus (IC) and the auditory cortex (AC). The non-auditory areas, including the hippocampus (HP), the amygdala (AMY) and the cerebellum (CRB), are also thought to be involved in tinnitus^4^,^20^. Alterations in the plasticity of the CNS may lead to enhanced neural activity in the CNS, which may result in tinnitus-like behaviour and persistent tinnitus.

Although many studies have investigated neural activity in the central auditory pathway (e.g., AC, IC, DCN, HP) during tinnitus^1^,^2^,^3^,^4^,^8^,^9^,, there are relatively few reports about the ultrastructural alterations. In this study, tinnitus was induced in rats via long-term salicylate administration, and tinnitus-like behaviour was confirmed using the gap pre-pulse inhibition of acoustic startle (GPIAS) and the pre-pulse inhibition (PPI) tests. A cerebral fluorine-18 fluorodeoxyglucose (18F-FDG) small animal positron emission tomography (microPET) system was used to identify the status of neural activity, whereas ultrastructural alterations of the synaptic endings were observed using transmission electron microscopy (TEM). As we previously reported, in contrast to the chronic effect of the long-term application of salicylate, DPOAEs^14^ and ASECA^11^ decreased after a single salicylate injection, with a minimum after ~2 h and then a progressive recovery over ~8 h; we also investigated the effects of single administration. The results of this study will help to elucidate the contribution of neuroplastic changes in the IC, AC and HP during reversible tinnitus induced by sodium salicylate.

## Results

### Salicylate-induced tinnitus-like behaviour in rats

**Figure 1:**
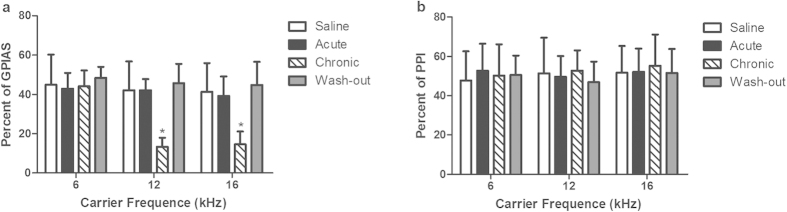
Results of gap pre-pulse inhibition of acoustic startle. (**a**) Effects of salicylate in gap pre-pulse inhibition of acoustic startle (GPIAS) values. The chronic treatment (n = 6 rats) group had a significant decrease in GPIAS values compared with the saline group (n = 6 rats) at 12 (chronic = 13.38 ± 4.61, saline = 42.19 ± 14.65, *p < 0. 05) and 16 kHz (chronic = 14.60 ± 6.50, saline = 41.33 ± 14.65,*p < 0.05), but not at 6 kHz (chronic = 44.18 ± 7.98, saline = 44.92 ± 15.34, *p* > 0.05). There were no significant differences in the GPIAS values among the acute treatment (n = 6 rats), wash-out (n = 6 rats), and saline groups (*p* > 0.05). (**b**) Effects of salicylate on pre-pulse inhibition (PPI) values. There were no significant differences in the PPI values among the four groups at 6, 12 and 16 kHz.

Salicylate-induced tinnitus was evaluated using the GPIAS test (n = 6 rats). A PPI test was performed in parallel to the GPIAS test to control that the GPIAS deficits are not due to temporal processing issues. Animals that fail GPIAS testing but pass PPI testing are thought to have tinnitus^21^,^22^. There were no significant differences in the PPI values among the four groups at 6, 12 and 16 kHz (Fig. 1b). Rats in the chronic treatment group showed a statistically significant decrease in GPIAS values relative to the saline group at 12 Hz (chronic = 13.38 ± 4.61, saline = 42.19 ± 14.65, *p* < 0.05) and 16 kHz (chronic = 14.60 ± 6.50, saline = 41.33 ± 14.65, *p* < 0.05) but not 6 kHz (chronic = 44.18 ± 7.98, saline = 44.92 ± 15.34, *p* > 0.05), indicating that these animals were experiencing tinnitus; tinnitus-like behaviour disappeared 14 days after the cessation of treatment (Fig. 1a). There were no significant differences in the GPIAS values among the other groups, indicating that the rats in the acute treatment group did not experience tinnitus (Fig. 1a,b).

### Mean standardized uptake values (SUVs) of 18F-FDG in each group

**Figure 2:**
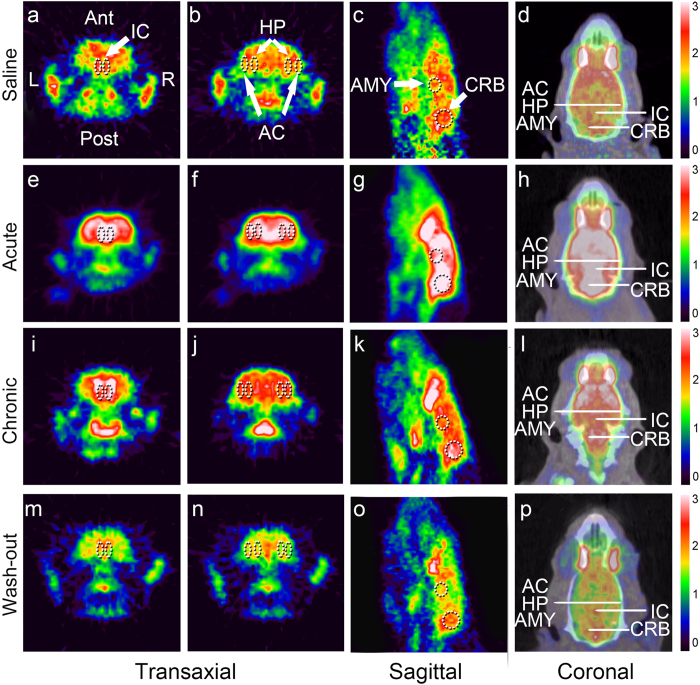
Corresponding transaxial (1^st^ and 2^nd^ columns) and sagittal (3^rd^ column) images obtained from microPET, fused CT/microPET (coronal, 4^th^ column) of a rat. (**a–d**) MicroPET brain images of rats from the saline group (n = 6 rats). (**e–h**) The mean SUVs in the acute treatment group (n = 6 rats) were significantly greater than those in the other three groups. (**i–l**) The mean SUVs in the chronic treatment group (n = 6 rats) were greater than those in the wash-out (n = 6 rats) and saline groups. (**m–p**) MicroPET brain images of rats from the wash-out group. There was no significant difference in the mean SUVs between the saline and wash-out groups. Dotted areas indicate the inferior colliculus (IC, 1^st^ column), auditory cortex (AC, 2^nd^ column, lateral areas), hippocampus (HP, 2^nd^ column, medial areas), amygdala (AMY, 3^rd^ column, upper areas), and cerebellum (CRB, 3^rd^ column, lower areas) at the corresponding levels (marked in the 4^th^ column). All rats were imaged in the prone position. PET images of average brain activity from the time of 18F-FDG injection until 40 min later are displayed with identical colour scales (right). Ant, anterior; Post, posterior; R, right; L, left.

**Figure 3:**
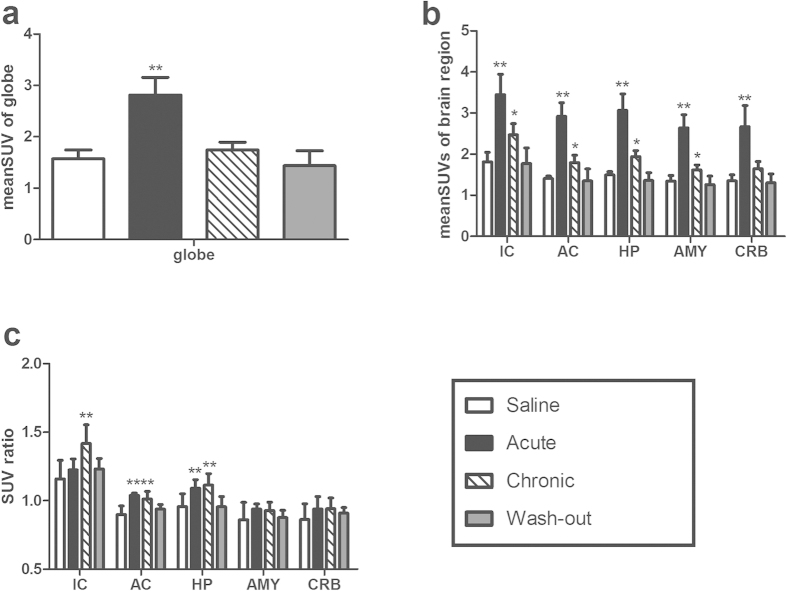
Mean SUVs of the whole brain (globe) and corresponding brain regions. (**a**) The mean SUVs of the globe. The mean SUVs of the globe in the acute treatment group were significantly greater (2.82 ± 0.34, n = 6 rats; *p* < 0.01) than those in the chronic treatment (1.75 ± 0.15, n = 6 rats), wash-out (1.44 ± 0.29 n = 6 rats), and saline (1.57 ± 0.17, n = 6 rats) groups. The chronic treatment group also showed higher mean SUVs, but the differences did not reach statistical significance (*p* > 0.05). (**b**) The mean SUVs of corresponding brain regions. The mean SUVs of the inferior colliculus (IC = 3.45 ± 0.50), auditory cortex (AC, 2.92 ± 0.33), hippocampus (HP, 3.07 ± 0.39), amygdala (AMY, 2.64 ± 0.32) and cerebellum (CRB, 2.67 ± 0.51) were significantly greater in the acute treatment group than in the chronic treatment, wash-out, and saline groups (**p < 0.01). The mean SUVs of the IC (2.47 ± 0.27), AC (1.80 ± 0.18), HP (1.94 ± 0.15) and AMY (1.62 ± 0.12), but not CRB (1.64 ± 0.18, *p* > 0.05) were greater in the chronic treatment group (*p < 0.05) than in the saline group. There were no significant differences in the SUVs between the saline and wash-out groups (*p* > 0.05). (**c**) The SUV ratio of corresponding brain regions. The SUV ratio of the AC (1.04 ± 0.02) and HP (1.09 ± 0.06) in the acute treatment group were significantly greater (**p < 0.01) than the saline group, suggesting relatively increased metabolism in the two brain regions of the rats in this group. The IC (1.22 ± 0.08), AMY (0.94 ± 0.04), and CRB (0.94 ± 0.09) did not show significant differences (*p* > 0.05). The SUV ratio of the IC (1.41 ± 0.14), AC (1.01 ± 0.05), and HP (1.11 ± 0.08), but not the AMY (0.93 ± 0.06; *p* > 0.05) or CRB (0.94 ± 0.08; *p* > 0.05), were greater in the chronic treatment group than in the wash-out and saline groups (**p < 0.01). There was also no significant difference in the SUV ratio between the saline and wash-out groups (*p* > 0.05).

Corresponding transaxial, sagittal and coronal microPET images of rats from all four groups are shown in Fig. 2. Dotted areas indicate the IC, AC, HP, AMY and CRB at the corresponding levels. All rats were imaged in the prone position. PET images of the average brain activity of rats were captured from the time of 18F-FDG injection until 40 min later. Visual comparisons of the images show evidence of increased FDG uptake in the IC, AC, HP, AMY and CRB as well as several other brain regions during the administration of sodium salicylate, which is in accordance with the results presented in Fig. 3.

The mean SUVs of the whole brain in the acute treatment group were significantly greater (2.82 ± 0.34, n = 6 rats; *p* < 0.01; Fig. 3a) than those in the chronic treatment (1.75 ± 0.15, n = 6 rats), wash-out (1.44 ± 0.29 n = 6 rats), and saline (1.57 ± 0.17, n = 6 rats) groups, indicating that the rats in the acute treatment group were in a state of high metabolic CNS activity. The chronic treatment group also had higher mean SUVs, but the differences did not reach statistical significance (*p* > 0.05, Fig. 3a). The mean SUVs of the IC (3.45 ± 0.50), AC (2.92 ± 0.33), HP (3.07 ± 0.39), AMY (2.64 ± 0.32), and CRB (2.67 ± 0.51) in the acute treatment group were significantly greater (*p* < 0.01; Fig. 2e–h; Fig. 3b) than those in the other three groups, and the mean SUVs of the IC (2.47 ± 0.27), AC (1.80 ± 0.18), HP (1.94 ± 0.15), and AMY (1.62 ± 0.12) but not CRB (1.64 ± 0.18, *p* > 0.05) were greater in the chronic treatment group than those in the wash-out and saline groups (*p* < 0.05; Fig. 2i–l; Fig. 3b). There was no significant difference in the SUVs between the saline (Fig. 2a–d; Fig. 3b) and wash-out groups (*p* > 0.05; Fig. 2m–p; Fig. 3b), which is in accordance with the GPIAS test results (Fig. 1a). These data indicate that metabolic activity decreases to baseline during recovery from tinnitus.

The SUV ratio (SUV ratio = mean SUV of brain region / mean SUV of globe) was calculated when the whole brain (globe) was used as a control region. The SUV ratios of the AC (1.04 ± 0.02) and HP (1.09 ± 0.06) were significantly greater in the acute treatment group (*p* < 0.01; Fig. 3c) than in the saline group, suggesting relatively increased metabolism in the two brain regions of the rats in this group. The IC (1.22 ± 0.08), AMY (0.94 ± 0.04), and CRB (0.94 ± 0.09) did not show significant differences (*p* > 0.05; Fig. 3c). The SUV ratios of the IC (1.41 ± 0.14), AC (1.01 ± 0.05), and HP (1.11 ± 0.08), but not of the AMY (0.93 ± 0.06; *p* > 0.05) or CRB (0.94 ± 0.08; *p* > 0.05), were greater in the chronic treatment group than in the wash-out and saline groups (*p* < 0.01; Fig. 3c), suggesting relatively increased metabolism in the three brain regions of rats during tinnitus. There was also no significant difference in the SUV ratio between the saline and wash-out groups (*p* > 0.05; Fig. 3c).

### Ultrastructural alterations of synaptic endings

**Figure 4:**
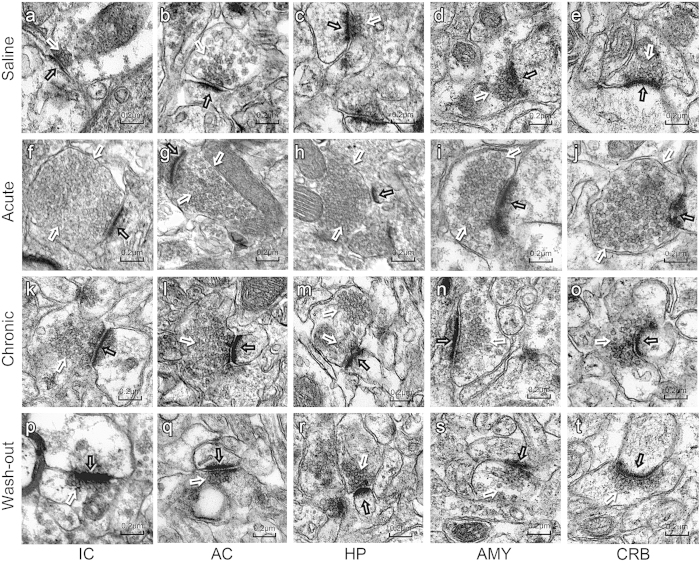
Ultrastructure of synaptic endings under TEM. (**a–e**) Synaptic ultrastructure of rats from the saline group (n = 3 rats). (**f–j**) Synapses of rats in the acute treatment group (n = 3 rats) contained a greater number of presynaptic vesicles (white arrows) than the other three groups. (**k–o**) Synapses of rats in the chronic treatment group (n = 3 rats) had more presynaptic vesicles (white arrows) in the inferior colliculus (IC), auditory cortex (AC), hippocampus (HP) and amygdala (AMY). There were greater PSDs (black arrows), longer synaptic active zones in the IC and AC, and increased synaptic interface curvature in the IC, AC and HP compared with the saline and wash-out (n = 3 rats) groups. No significant ultrastructural changes were observed in the cerebellum (CRB). **(p–t)** Synaptic ultrastructure of rats from the wash-out group; there were no significant differences observed between the saline and wash-out groups. Scale bar, 0.2 μm.

Twelve rats (three from each group) were used for the ultrastructural study. Quantitatively, the ultrastructure of the IC and AC neurons contained an increased number of synaptic vesicles (IC, Fig. 4k; AC, Fig. 4l; *p* < 0.01), with greater postsynaptic densities (PSDs) (IC, Fig. 4k; *p* < 0.01; AC, Fig. 4l; *p* < 0.05), longer synaptic active zones (IC, Fig. 4k; *p* < 0.01; AC, Fig. 4l; *p* < 0.05) and an increased synaptic interface curvature (Fig. 4k, l; *p* < 0.05) in the chronic treatment group compared with the saline group. The ultrastructure of the HP neurons contained an increased number of synaptic vesicles (Fig. 4m; *p* < 0.01) in the chronic salicylate-treatment groups, with increased an synaptic interface curvature (Fig. 4m; *p* < 0.05) compared with the saline group. More synaptic vesicles were only observed in the AMY (Fig. 4n; *p* < 0.01) neurons, and no significant ultrastructural changes were observed in the CRB (Fig. 4o; *p* > 0.05). These results indicate the increased speed and efficacy of chemical synaptic transmission in the three brain regions (IC, AC and HP) during tinnitus. The ultrastructure of the IC, AC, HP, AMY and CRB neurons showed a remarkably increased number of synaptic vesicles (Fig. 4f–j; *p* < 0.01), but no other changes were observed in the acute salicylate-treatment group compared with the saline group. Combined with the PET scan results, these results indicate an acute excitatory state of these brain regions. There were no significant differences between the saline (Fig. 4a–e) and wash-out groups (Fig. 4p–t). These results are summarized in Table 1.

## Discussion

The mechanisms and suspected origins of tinnitus have not yet been fully elucidated^1^,^2^,^3^,^4^. Subjective idiopathic tinnitus is a symptom that is not routinely detected during clinical and paraclinical examinations, which renders it difficult to analyse the causes of this condition. Tinnitus can arise from damage at any level of the auditory pathway, but most cases are caused by cochlear damage. These lesions can result in neuroplasticity alterations in the peripheral and central auditory systems^1^,^4^,^14^. An increasing number of electrophysiological and neuroimaging studies have revealed that tinnitus is linked with increased activity in the central auditory system^9^,^16^,^23^,^24^. Moreover, abnormal neural activity might be interpreted and perceived as tinnitus in higher cortical centres (e.g., IC, AC, as well as associated cortices and nuclei)^25^,^26^. However, few studies have examined the *in vivo* neural activity during tinnitus. In the auditory system, the AC is the most highly organized processing unit of sound in the brain, and major ascending auditory pathways converge in the IC, appearing as an integrative station and switchboard, respectively. Severe tinnitus is often accompanied by affective disorders, such as stress, depression and emotional processes. This has led to the view that tinnitus is a complex brain disorder, with various brain regions mediating perception, distress, salience, memory and attention, including the HP, AMY and CRB. However, the relationship between these regions remains unclear^4^,^20^. Therefore, we developed a tinnitus rat model via long-term salicylate administration and confirmed tinnitus-like behaviour using the GPIAS test. Then, cerebral 18F-FDG microPET was used to measure metabolic activity in the IC, AC, HP, AMY and CRB, whereas ultrastructural alterations in the synapses were observed using TEM. The aim of this study was to prove the hypothesis of tinnitus centralization in the CNS. More specifically, we expected to find neuroplasticity changes related to metabolic activity and ultrastructure within auditory and non-auditory structures.

PET imaging of 18F-FDG is widely used to image neural activation in the brain^9^,^27^,^28^,^29^. In previous human PET imaging studies of noise- and age-induced tinnitus, increased or asymmetric changes in metabolic activity in the CNS were detected^30^,^31^. Altered activity was also detected in brain regions, including the AC and surrounding areas, such as the medial geniculate body, temporal-parietal auditory association areas, and limbic regions (i.e., HP and AMY). Due to the improved resolution of microPET scanners, 18F-FDG PET imaging has become a useful approach to monitor cerebral metabolic patterns^27^,^28^,^29^ and identify the central auditory structures involved in tinnitus^9^. Animal models have thus far only been used to document the perceptual auditory aspect of tinnitus, not its emotional impact. Thus, we investigated areas in the central auditory pathway as well as non-auditory areas (HP, AMY and CRB) to reveal the relationship between these areas and tinnitus.

Synaptic plasticity, which is one of the most important aspects of neuroplasticity, includes both short-term changes in the strength and efficiency of neurotransmission and long-term changes in synaptic structures^32^. An increase in the number of synaptic vesicles may result in increased neurotransmitter release and subsequent synaptic transmission. A previous study reported huge lucent pleomorphic vesicles in the same area of the chinchilla following acoustic trauma^33^. The correlated length of the synaptic active zone, synaptic curvature, synaptic interface, and PSD is thought to be important for the speed and efficacy of chemical synaptic transmission^32^. In our previous studies of salicylate-induced tinnitus, synaptic ultrastructure alterations in the DCN were observed^13^,^19^. We were also interested in the other brain areas involved in tinnitus. Hoping to determine whether the increased metabolic activity was accompanied by ultrastructural changes, we observed ultrastructure of synaptic endings using simultaneous TEM.

In this study, we found that activation of the IC, AC, and HP was upregulated in rats with tinnitus. Using TEM, we observed a greater number of presynaptic vesicles, and most of the PSD appeared greater, while some were longer, with a greater curvature of the synaptic interfaces. These fine structural changes to the synapse may reflect an increased speed and efficacy of chemical synaptic transmission, which might lead to enhanced neural activity. Our results from microPET scans also showed enhanced glucose uptake, a measure of metabolic/neuronal activation, in the IC, AC, and HP following the induction of tinnitus. The ultrastructural findings were in accordance with the microPET results. There were fewer alterations in the AMY and CRB, but there was also a slightly increased FDG uptake. In our previous studies, neuroplasticity-related receptor and immediate to early gene expression of NR2B and Arg3.1 were upregulated in the DCN after chronic salicylate treatment, but they returned to normal after a 14-day recovery period^13^. These factors might be responsible for the observed ultrastructural changes, as all were almost synchronized with the appearance of tinnitus symptoms, and all were ameliorated 14 days after the cessation of treatment. These fluctuations over time indicate that the nervous system continually attempts to rebalance these changes during the process. Hence, we believe that neuroplasticity of the CNS plays an important role in the response to salicylate treatment.

We found no behaviour-related signs of tinnitus in the acute salicylate-treatment group. The mean SUVs of the IC, AC, HP, AMY and CRB were significantly greater than those of other groups, and there were no other ultrastructural changes. However, there was an increased release of presynaptic vesicles, which contain a large quantity of neurotransmitters. The tinnitus-related hyperactivity observed in the central auditory pathway may be due to a reduction in γ-aminobutyric acid (GABA)-mediated inhibition^12^,^25^,^34^ or an increase in glutamatergic activation^13^,^19^,^20^,^35^. It has been shown that GABAergic and glutamatergic neurotransmission can mediate functional alterations in the central auditory pathways^36^. In the long-term administration of salicylate, the repeated release of a great number of presynaptic vesicles may result in greater PSD, a longer synaptic active zone, and an increased synaptic interface curvature to meet the need for increased speed and efficacy of chemical synaptic transmission, as was observed in the chronic treatment group. N-methyl-D-aspartic acid (NMDA) receptors, members of the glutamate receptor channel superfamily, are located mainly in the PSD of neurocytes. It was reported that salicylate induces tinnitus through activation of cochlear NMDA receptors, which may act on cochlear fast synaptic transmission^37^,^38^. In our previous studies, the expression of the NMDA receptors was increased in the DCN^13^,^19^, IC and AC^35^ during salicylate-induced tinnitus, which may be responsible for the greater PSDs. Our previous study demonstrated that a single injection of salicylate reduced the amplitude of DPOAEs with decreased OHC electromotility, which is in contrast to the long-term treatment effects^14^,^17^. Hence, we consider the response to acute salicylate-treatment to be a transient stress response rather than a continuous response. Sztuka *et al.*^39^ reported that hyperacusis was found in 63% of the tinnitus patients with no hearing loss. Their results indicated that hyperacusis influences DPOAE amplitude and increases its value, which was similar to our chronic model. There are still problems and limitations of GPIAS test. However, hearing loss, temporal processing deficits, and hyperacusis remain confounding variables when using traditional gap detection paradigms to measure tinnitus in animals^40^. Since an animal cannot directly communicate its subjective experiences, there is a lack of reliable animal behavioral models of these disorders. The high prevalence of hyperacusis in the tinnitus population suggests a common mechanism of dysfunction for these two perceptual disorders^41^. These ultrastructural synaptic changes in chronic treatment group indicate a hyper-reactivity, and may form the structural foundation of tinnitus centralization by activating the central auditory system. Which of the neural correlates in the chronic treatment group are due to tinnitus, which are due to hyperacusis ? This question is hard to answer. We also found that the degree of ultrastructural changes in the five areas differed. Changes in the IC were the most significant, whereas those in the CRB were the least significant. The roles of non-auditory areas during tinnitus are in doubt, and the transition from acute to chronic tinnitus remains unclear. Further research is needed to elucidate the mechanisms underlying these processes.

Salicylate is a well-known ototoxic drug that can cause reversible tinnitus and hearing loss. In addition to acting on OHCs to influence hearing function, it can also penetrate the blood-brain barrier and directly interfere with neural activity at sites within the central auditory pathway^3^,^8^. In our previous studies, we developed a tinnitus rat model using the long-term administration of relatively low-dose salicylate (200 mg/kg) for single injections twice daily for 14 consecutive days. This model induced alterations of the ASECA and very small hearing loss without modifications to the compound action potential (CAP)^11^,^14^. The model was remarkably similar to the characteristics of salicylate-induced tinnitus in humans. However, a single injection of sodium salicylate was shown to reduce the ASECA and DPOAEs after 2 h, and recovery was achieved by 8 h^14^. Together, these findings support the proposition that the development of tinnitus is long-term, and the reactions of the auditory system to single and repeated salicylate treatment differ. Several previous studies have indicated that an increase in AC neuronal activity may be an indicator of tinnitus^9^,^24^,^42^, which is consistent with our results. However, several controversial issues remain to be addressed. For example, a former study indicated that high-dose salicylate administration was associated with a significant increase in metabolic activity in the AC and IC, but to a lesser extent in the thalamus^9^. In their study, the rats received salicylate (250 mg/kg) for 2 days to induce tinnitus, and schedule-induced polydipsia avoidance conditioning was then employed to evaluate the degree of tinnitus, which was different from that used in the present study. MicroPET scans were implemented before and following salicylate treatment. A gerbil model was used in another study to evaluate the metabolic activity of the AC and subcortical structures using [carbon-14] 2-deoxyglucose autoradiography after treatment with salicylate (200–350 mg/kg) for 4 days^42^. However, the results indicated that glucose uptake was decreased in the IC and other subcortical structures, including the cochlear nucleus and lateral lemniscus, in the salicylate-treatment group compared with the saline-treatment group. These results suggest that salicylate-induced tinnitus activates the AC without activation of the IC. Other clinical reports have indicated that tinnitus often occurs several days to weeks after the administration of aspirin (salicylate), and the severity of symptoms increases (becomes louder) as treatment is continued^43^. The administration of high-dose salicylate reliably induces hearing loss and tinnitus, although there was no tinnitus-like behaviour among rats in the acute treatment group in the present study. The process of developing persistent tinnitus and the accompanying cortical reorganization that alters neural synchrony are both prolonged and slow^15^. The amount of time required for salicylate to induce tinnitus remains unclear. We chose different time points for our experimental tests (2 h after single injection, 14 days after treatment and 14 days for the recovery period). Differences between species, duration and dosage of salicylate treatment, assessment methods, and other study techniques may be responsible for the differences between the results of previous studies and the present study.

Maladaptation of central information processing is thought to be responsible for tinnitus perception and generation^7^,^44^. Human neuroimaging and neurophysiological evidence supports this notion by implicating the central auditory system and the prefrontal and emotional centres in tinnitus, indicating that tinnitus is a complex brain disorder across distributed auditory and non-auditory brain regions^1^,^2^,^3^,^4^,^45^,^46^,^47^,^48^. These regions are considered to mediate perceptual, attentional, and emotional processes, and changes in the auditory pathways are likely to trigger alterations within these processes^49^,^50^. Structural abnormalities were detected in the subgenual anterior cingulate cortex^51^,^52^ and the HP^53^ following salicylate administration. Symptoms, such as frustration, annoyance, irritability, anxiety, and depression, are closely related to tinnitus^1^,^2^,^4^,^48^. However, it remains uncertain to what extent the observed changes in non-auditory areas are genuinely related to tinnitus perception, or to what extent they might only reflect the impact of tinnitus distress. Co-activation of the hippocampal–cortical memory system may be associated with the development of persistent tinnitus^4^. Interplay between the HP and posterior inferior temporal cortex was shown in an experiment investigating memory consolidation during acute stress^54^. The results of the study showed that tinnitus distress was associated with a concomitant increase in glucose metabolism in the HP areas, which suggests a mechanism of maladaptive memory consolidation. Taken together, these findings indicate that salicylate affects both the cochlea and the central auditory pathway and also has widespread effects on the non-auditory regions of the CNS.

In addition to direct action on the cochlea and CNS, salicylate overdose is also known to cause abdominal discomfort in some animals^55^, which may cause negative emotional and health effects. The onset of tinnitus can also be associated with emotional factors and stress, and any combination of altered auditory and somatosensory inputs together with abnormal activity in the central nervous structures can be relevant for tinnitus development^1^,^2^,^4^. There has been disagreement about whether tinnitus may result from increased activity in the cochlear nerve or cochlear insults. However, the tinnitus field has undergone a major paradigm shift, as a central origin of tinnitus-related activity seems to have replaced the former peripheral hypothesis^1^,^2^,^4^,^7^,^15^. Tinnitus could be triggered by aberrant peripheral spontaneous activity, which may be abnormally amplified by central auditory structures and propagate throughout the brain regions involved in conscious perception. Therefore, we hypothesized that a single high-dose treatment with salicylate may act as a stressor and induce the release of an abundance of neurotransmitters in auditory-associated and other areas of the CNS (such as the IC, AC, and HP). The effects of long-term salicylate treatment, as well as peripheral auditory injury, might be interpreted as chronic abnormal stimulation by the CNS, which may induce ultrastructural changes to compensate and therefore maintain a stable state. The subsequent upregulated neuronal excitability corresponded to an upregulation in the metabolic rate and may be responsible for the behaviour-related signs of tinnitus in the chronic salicylate-treatment group. Maladaptation of central information processing, ultrastructural changes, and functional reorganization then take place in some areas of the cerebrum that may lead to persistent tinnitus. Although animal models provide an important opportunity to gain insight into the neuronal mechanisms of tinnitus, research on tinnitus-related emotional or cognitive symptoms is still preliminary and inconclusive, and it remains unclear which aspects of tinnitus in humans are truly reflected in the existing animal models^1^,^4^. There is still a great need for analysing tinnitus-related brain changes in human studies due to the limitations of animal studies.

The data we report show that chronic salicylate-induced tinnitus in rats is associated with ultrastructural alterations to the synapses and a significant increase in metabolic activity in the IC, AC, and HP. We found that tinnitus duration and distress were correlated with activation of the brain areas outside the classical central auditory pathways. Together, these findings suggest a mechanism for tinnitus centralization accompanied by homeostatic plasticity following peripheral auditory injury, and neuroplasticity might be responsible for these changes. These results will help to further elucidate the role of neuroplasticity in several areas of the CNS affected by tinnitus.

## Methods

### Animal preparation and experimental design

Twenty-four male Sprague–Dawley rats (weight, 200–350 g) were obtained and maintained under specific pathogen-free conditions. The animals were randomly divided into four groups: (1) an acute treatment group that received a single injection of salicylate 2 h before the test (n = 6 rats), (2) a chronic treatment group that received repeated injections of salicylate for 14 days (n = 6 rats), (3) a wash-out group that received repeated injections of salicylate for 14 days and was allowed to recover for 14 days (n = 6 rats), and (4) a saline group that received repeated injections of saline (n = 6 rats). All experimental procedures involving the animals were approved by the Institutional Review Board of Xinhua Hospital and were performed according to the guideline of the National Institutes of Health for the care and use of laboratory animals (NIH publication No 80-23).

### Salicylate administration

The tinnitus model was created based on our previous studies, and details of the methods can be found in our earlier publications^11^,^14^,^17^,^19^. Sodium salicylate (Sigma–Aldrich, Shanghai, China) was dissolved in saline to achieve a concentration of 200 mg/ml. The acute treatment group received a single intraperitoneal injection of salicylate (400 mg/kg) 2 h before the GPIAS test and PET scan. The chronic treatment group received twice-daily intraperitoneal injections (200 mg/kg) at 08:00 and 16:00 h for 14 consecutive days, whereas the saline group received intraperitoneal injections of saline solution (200 mg/kg) at the same time points, and the wash-out group received intraperitoneal injections for 14 consecutive days followed by a 14-day recovery period. The chronic treatment and saline groups underwent the GPIAS test and PET scans on day 15. The wash-out group was tested on day 29. After the PET scans, 12 rats (three from each group) were used for the TEM study.

### Gap pre-pulse inhibition of acoustic startle

Tinnitus was assessed using the GPIAS and PPI paradigm as described in detail in previous reports^13^,^22^,^56^,^57^. This programme exploits the acoustic startle reflex in animals treated with salicylate. GPIAS and PPI testing was performed using the Acoustic Startle Reflex Starter Package for Rat or Mouse (Med Associates, St. Albans, VT, USA), which consists of a sound-attenuating cubicle, a stimulus panel with a platform table and speakers, a load-cell platform, and an amplifier. An acrylic animal holder for the startle response was mounted interchangeably on the load-cell platform. Animal holder movement resulted in the displacement of the load cell, generating a voltage proportional to the velocity of displacement. This analogue output was amplified and digitized using Med Associates hardware interfaced to a PC. The stimulus panel included two speakers for presentation of the background white noise and acoustic startle stimuli. The GPIAS and PPI testing began 1 h before the rats were scanned. The peak-to-peak values of the amplitude of the startle response were collected by a PC and analysed offline.

GPIAS sessions were composed of 30 gap trials and 30 no-gap trials. The rats underwent testing with different band-pass-filtered sounds centred at 6 kHz, 12 kHz or 16 kHz at a sound pressure level (SPL) of 65 dB. Startle responses were elicited by a 20-ms burst of white noise at a SPL of 100 dB. The gap in the narrowband noise began 100 ms before the onset of the broadband startling noise and lasted for 50 ms (5 ms rise/fall time). The interval between each startling noise was 30–35 s, and each test took approximately 30 min. PPI sessions were composed of 30 startle trials and 30 pre-pulse trials. The startle stimulus was either presented alone or was preceded by a 65 dB SPL narrowband noise-burst (50 ms, 5 ms rise/fall time) pre-pulse centred at 6 kHz, 12 kHz or 16 kHz, the same frequencies and levels used for GPIAS testing. The onset of the noise burst pre-pulse preceded the onset of the startle stimulus by 100 ms. The PPI testing was immediately done after the GPIAS testing and each test took approximately 30 min.

The percent of GPIAS was calculated by computing the average ratio of the gap versus no-gap trials for each frequency using the formula: [(AvgTnogap – AvgTgap) / AvgTnogap × 100%], where AvgTgap is the average amplitude during the gap trials, and AvgTnogap is the average amplitude of the no-gap trials. The percent of PPI was calculated by computing the average ratio of the startle versus pre-pulse trials for each frequency using the formula: [(AvgTstartle – AvgT pre-pulse) / AvgTstartle × 100%], where AvgTpre-pulse is the average amplitude during the pre-pulse trials, and AvgTstartle is the average amplitude of the startle trials^24^,^58^.

### 18F-FDG microPET

The Department of Nuclear Medicine at the PET Center of Huashan Hospital (Fudan University) provided the 18 F-FDG used in this study. Prior to the scan, the rats were all fasted (but with free access to water) for 8–12 hours to maximize FDG uptake in the brain. To minimize FDG uptake in the skeletal muscle and brown adipose tissue, the rodents were kept in a warming system. During the scan, a heating blanket was used to maintain the ambient temperature in the PET/CT scanner.

Each rat was anaesthetized with a gas mixture of isoflurane (1%) and oxygen (1 L/min) approximately 5 min before scanning in an anaesthetizing box and then subsequently in an inhalation system installed on the scanner that covered the rodent’s mouth and nostrils with a mask. The body weight and radioactivity of the syringe (both before and after injection) were measured for calibration of the effective dosage.

The PET signals and hybrid CT data were acquired with a Inveon MM micro-PET/CT scanner (Siemens Co., Ltd, Knoxville, TN, USA) after an awakening uptake period of approximately 45 min after the intravenous 18F-FDG injection through the caudal vein. The 20-min listmode PET data were binned into one single frame and were reconstructed using an OSEM3D/MAP algorithm and attenuation correction information derived from the CT. The iterations of OSEM3D = 2, and the iterations of MAP = 18. The final voxel size and the matrix were 0.776 mm × 0.776 mm × 0.796 mm and 128 × 128 × 159, respectively.

The PFUS module of the PMOD 3.4 (PMOD Technology, Switzerland) was applied to analyse the images. A Gaussian kernel with a full width of half maximum (FWHM) of 0.8 mm × 0.8 mm × 0.8 mm was used to smooth the brain image during the normalization process after automatic rigid matching of the individual rat brain to the template. Afterwards, a Schiffer atlas^59^ was overlaid onto the normalized individual PET images to obtain the SUVmean of all 58 rat brain regions in the standard template. The parameter SUV was defined as: [tissue activity concentration (kBq/cc) × body weight (g) / effective injected radioactivity (kBq)]. Eventually, each regional SUVmean was divided by that of the global average, as an SUV ratio, for statistical analysis.

### Transmission electron microscopy

Twelve rats (three from each group) were used for the TEM study after PET scans. Briefly, anaesthetized rats were perfused with 2% glutaraldehyde through the ascending aorta. After perfusion, the brains were removed from the skull, and tissue blocks containing the IC, AC, HP, AMY and CRB were dissected and washed in 0.1 M phosphate buffer (pH 7.2). The tissue samples were then immersed in 2% glutaraldehyde and 1% osmium tetroxide for 2 h at 4 °C. Subsequently, the tissue blocks were routinely dehydrated using graded ethanol. After displacing the ethanol with propylene oxide, the tissues were embedded with Epon solution. A consecutive series of ultrathin (70 μm) sections in the coronal plane were prepared using a diamond knife and then stained with lead citrate and observed under a CM-120 TEM (Philips Innovation Services, Eindhoven, the Netherlands). ImageJ software was used for quantitative analysis of six sections of each tissue block for each of the two hemispheres. The number of synaptic vesicles, PSD thickness, synaptic cleft width, synaptic interface curvature, and length of the synaptic active zone were measured in each photo taken at 33,000 × magnification^60^.

### Statistical analysis

Results are expressed as the means ± standard errors of the means. According to the distribution of the data and homogeneity of variance, an unpaired, two-tailed Student’s *t*-test and one-way analysis of variance followed by the Student–Newman–Keuls *post-hoc* test were used for comparisons among groups, except that GPIAS and PPI testing were tested for significance with a two-way analysis of variance (frequency and treatment groups) followed by the Tukey *post-hoc* test. A probability (*p*) value of < 0.05 was considered statistically significant. All statistical analyses were performed using SPSS version 18.0 software (IBM-SPSS, Inc., Chicago, IL, USA).

## Additional Information

**How to cite this article**: Yi, B. *et al.* Effects of long-term salicylate administration on synaptic ultrastructure and metabolic activity in the rat CNS. *Sci. Rep.* **6**, 24428; doi: 10.1038/srep24428 (2016).

## Figures and Tables

**Table 1:** Comparisons of synaptic parameters among different groups.

Area	N = 18	Saline	Acute	Chronic	Wash-out
IC	Synaptic vesicles (number/μm^2^)	18 ± 7	130 ± 31**	60 ± 25**	20 ± 7
	Cleft width (nm)	17.42 ± 3.29	19.71 ± 5.68	16.99 ± 5.62	19.29 ± 2.06
	PSD thickness (nm)	22.38 ± 9.37	29.12 ± 7.37	41.24 ± 9.44**	27.94 ± 5.41
	Synaptic curvature	1.07 ± 0.01	1.04 ± 0.02	1.10 ± 0.04*	1.05 ± 0.03
	Length of synaptic active zone (nm)	338.73 ± 25.99	303.08 ± 73.12	504.19 ± 91.40**	334.96 ± 83.01
	Synaptic vesicles (number/μm^2^)	18 ± 8	94 ± 27**	50 ± 13**	17 ± 4
	Cleft width (nm)	15.51 ± 2.07	20.15 ± 4.20	16.47 ± 3.26	20.11 ± 4.11
AC	PSD thickness (nm)	29.18 ± 6.81	31.13 ± 4.81	38.70 ± 8.68*	27.67 ± 6.01
	Synaptic curvature	1.04 ± 0.04	1.08 ± 0.02	1.14 ± 0.06*	1.05 ± 0.04
	Length of synaptic active zone (nm)	279.52 ± 57.04	297.86 ± 44.72	400.07 ± 97.15**	314.56 ± 57.36
	Synaptic vesicles (number/μm2)	17 ± 6	68 ± 24**	37 ± 13**	13 ± 3
	Cleft width (nm)	18.95 ± 2.76	18.33 ± 3.31	16.50 ± 3.34	18.95 ± 2.81
HP	PSD thickness (nm)	34.22 ± 5.67	29.43 ± 7.46	33.25 ± 5.51	38.88 ± 9.67
	Synaptic curvature	1.05 ± 0.03	1.07 ± 0.02	1.14 ± 0.04*	1.05 ± 0.03
	Length of synaptic active zone (nm)	240.81 ± 64.02	231.98 ± 75.29	272.53 ± 56.78	244.99 ± 42.69
	Synaptic vesicles (number/μm^2^)	16 ± 4	64 ± 10**	27 ± 7**	14 ± 3
	Cleft width (nm)	19.13 ± 2.04	18.33 ± 3.79	17.53 ± 2.63	18.49 ± 2.81
AMY	PSD thickness (nm)	28.81 ± 7.33	26.18 ± 7.61	33.93 ± 6.47	30.43 ± 5.10
	Synaptic curvature	1.05 ± 0.03	1.06 ± 0.03	1.09 ± 0.03	1.06 ± 0.02
	Length of synaptic active zone (nm)	337.69 ± 78.99	344.45 ± 99.20	356.13 ± 70.88	329.98 ± 68.91
	Synaptic vesicles (number/μm^2^)	18 ± 5	71 ± 16**	23 ± 11	20 ± 6
	Cleft width (nm)	19.02 ± 2.61	19.42 ± 3.52	17.47 ± 3.76	18.54 ± 2.43
CRB	PSD thickness (nm)	34.08 ± 5.68	32.82 ± 6.77	31.30 ± 5.00	30.71 ± 4.84
	Synaptic curvature	1.05 ± 0.03	1.07 ± 0.04	1.07 ± 0.03	1.06 ± 0.02
	Length of synaptic active zone (nm)	312.48 ± 100.84	317.34 ± 60.80	330.97 ± 61.58	302.98 ± 93.49

N, number of photographs. Saline group (n = 3 rats), acute treatment group (n = 3 rats), chronic treatment group (n = 3 rats), wash-out group (n = 3 rats). Inferior colliculus (IC), auditory cortex (AC), hippocampus (HP), amygdala (AMY), and cerebellum (CRB). *p < 0.05 compared with saline group; **p < 0.01 com*p*ared with saline group.
